# Point-Of-Care or Point-Of-Need Diagnostic Tests: Time to Change Outbreak Investigation and Pathogen Detection

**DOI:** 10.3390/tropicalmed5040151

**Published:** 2020-09-25

**Authors:** Sören Hansen, Ahmed Abd El Wahed

**Affiliations:** 1 Division of Microbiology and Animal Hygiene, University of Goettingen, Burckhardtweg 2, D-37077 Goettingen, Germany; hansensoer@gmail.com; 2 Institute of Animal Hygiene and Veterinary Public Health, University of Leipzig, An den Tierkliniken 43, D-04103 Leipzig, Germany

**Keywords:** point-of-care, point-of-need, diagnostics, lateral flow assay, isothermal amplification, mobile laboratory, field applicable diagnostics

## Abstract

In the recent years, the progress of international trade and travel has led to an increased risk of emerging infections. Around 75 percent of the pathogens causing these infections are of animal origin. Point-of-care tests (POCT) and point-of-need tests (PONT) have been established in order to directly provide accurate and rapid diagnostics at field level, the patient bed-side or at the site of outbreaks. These assays can help physicians and decision makers to take the right action without delay. Typically, POCT and PONT rely on genomic identification of pathogens or track their immunological fingerprint. Recently, protocols for metagenomic diagnostics in the field have been developed. In this review, we give an overview of the latest developments in portable diagnostic methods. In addition, four mobile platforms for the implementation of these techniques at point-of-care and point-of-need are described. These approaches can provide reliable diagnostics and surveillance, especially in low resource settings as well as at the level of one health.

## 1. Background

Many different microorganisms such as bacteria, viruses, fungi, or parasites exist in the environment. Most of them are important to maintain the ecosystem on earth, however, some of them are pathogenic and cause infectious diseases in both humans and animals. Infectious diseases are one of the major reasons for death especially in the low- and middle-income regions of the world [1]. 

While globalization and international travel progresses, the risk of emerging diseases is increasing. Due to global flight connections, people, goods, or traded animals can reach almost every location in the world within one or two days. Unfortunately, this time frame is shorter than the incubation periods of several important zoonotic and animal diseases with an emerging potential defined by the World Organization for Animal Health (OIE, Table 1, [2]).

Around 75 percent of all new emerging diseases are zoonotic [3]. In many African countries, one of the causes leading to the emergence of pathogens is bush meat, since people living in rural areas depend on it as nutrition source. Not only the consumption but also the processing of the meat can lead to an infection [4]. Also, settlements in forests and deforestation are driving factors in the spread of infectious diseases as the habitats of pathogens´ vectors and reservoirs shrink and start to overlap with areas where domesticated animals and people live [5]. The danger of being infected by a zoonotic disease is not limited to low resource settings. In general, every person who lives or works in close contact to animals is at risk. This is particularly true for people living on a farm or together with pets but also for people in contact with wild or zoo animals [6]. Unfortunately, there is a lack of diagnostic capacity in many regions of the world. Thus, disease outbreaks may stay undetected for longer time and can spread through the population of the affected area. For instance, in 2013, only twelve countries in sub-Saharan Africa have laboratories accredited to international standards. Ninety-one percent of the 380 laboratories were located in South Africa [7]. These numbers show the urgent need for diagnostic capacity in sub-Saharan Africa. 

Another example for the need of sophisticated diagnostics are natural disasters as they are often followed by infectious disease outbreaks. Normally an outbreak starts during the post-impact phase within the first four weeks after the disaster. Depending on the type of the catastrophe, the resulting disease differs. For instance, floods more often lead to mosquito-borne infections, while earthquakes are correlate with diseases occurring due to contaminated food or water sources [8]. The World Health Organization (WHO) defines a relatively-small number of diseases related to natural disasters. In contrast, interviews with experts involved in previous disaster situations revealed that there are several important pathogens, which are listed in Table 2 [9,10].

A profound surveillance system is one of the main factors in preventing the spread of infectious diseases [11], which can only be achieved with highly-advanced diagnostics. The current diagnostic approaches rely on centralized reference laboratories with high throughput, due to the need of complex and expensive devices. Point-of-care testing (POCT) describes the identification of pathogens near the patient with a fast turn-around time and the potential to immediately change in the health management [12]. While the term POCT is used for human patients and samples, point-of-need testing (PONT) has a broader meaning and also includes on-site testing of environment, animals, and food samples, although this term is not yet clearly defined. The WHO formulated the characteristics of POCT and PONT as affordable, sensitive, specific, user friendly, robust and rapid, equipment-free, and deliverable to those who need them (ASSURED criteria, [13]). 

Examples for rapid diagnostics in the field are the POCTs for malaria and human immunodeficiency viruses (HIV). As a consequence of rapid testing, disease control as well as change in treatment and care was achieved particularly, in regions where stable electricity supply, highly trained personal, or specialized devices are not available [14]. Most POCT and PONT rely on immuno-techniques to detect antigens or antibodies, but methods for the identification of the pathogen at molecular level are on the rise [15]. In this review, we provide an overview of the latest field-applicable diagnostic methods and techniques as well as their implementation at point-of-care (POC) and point-of-need (PON).

## 2. Immunoassays for Identification of Pathogens and Antibodies

**Figure 1:**
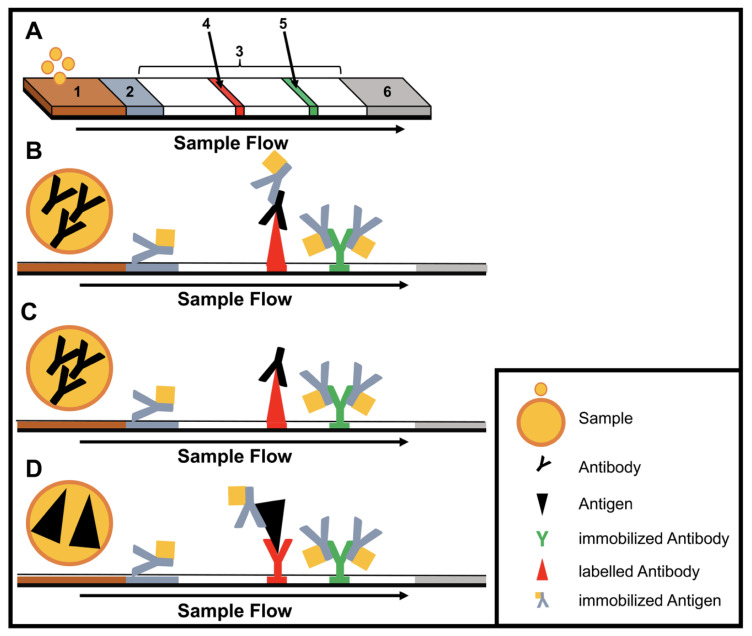
Structure and type of a lateral flow immunoassay. (**A**) Schematic design: 1, sample pad; 2, conjugate pad; 3, membrane; 4, test line; 5, control line; 6, adsorbent pad. (**B**) Indirect lateral flow immunoassay. (**C**) Competitive lateral flow immunoassay. (**D**) Sandwich lateral flow immunoassay. The principle of a lateral flow immunoassay (LFIA) is as follows—the sample is brought onto the sample pad and flows in the opposite direction by adsorption. While the sample passes the conjugate pad, a labelled target-specific antibody binds to the target molecule in the sample (antigen or antibody). Afterwards, the labelled antibody–target complex is immobilized to the membrane by a specific capture molecule (antibody or antigen) adhered to the membrane at the test line. The unbound labelled antibodies are captured at a control line by immobilized antibodies. In the case of a positive sample, the accumulation of the labelled antibodies leads to a coloration of both test lines. In the case of a negative sample, only the control line is colored. The adsorbent pad takes up the excess liquid. In the case of pathogens or targets, which are not suitable for the indirect or sandwich designed LFIAs, a competitive layout can be applied. This results in only one colored test line in case of a positive test.

Immunoassays are based on binding of an antibody and an antigen to each other. The simplest and most used equipment-free assays are lateral flow immunoassays (LFIAs) as they are performed in a small disposable cartridge (Figure 1). LFIAs do not require pipetting or washing steps and can be performed by untrained personal. In addition, no cold chain is required. Labelling of the secondary antibodies is accomplished mostly by gold or silver nanoparticles. LFIAs are able to detect pathogen-specific antigens and/or antibodies within 10–30 min. Recently, multiplex LFIAs have been developed for detecting multiple targets in a single test [16]. 

Paper-based micro fluid devices (µPAD) are yet another development of the lateral flow technique. Here, the sample is guided by hydrophobic channels [17]. This design enables more sophisticated applications like the combination of multiple serological assays in a single device [18,19].

A critical drawback of this technology is the sensitivity and specificity, as lateral flow assays (LFA) highly depend on the labelling and binding affinity of the tested biomolecules [20]. A high concentration of molecules is necessary to achieve positive results. Therefore, LFA will produce a false negative result, when testing samples containing only few target molecules. Moreover, on some occasions sample preparation is necessary since the test design only allows liquid non-viscous samples. Also, quantification of targets is not possible using the LFA.

## 3. Methods for the Identification of Pathogens at the Genomic Level 

**Figure 2:**
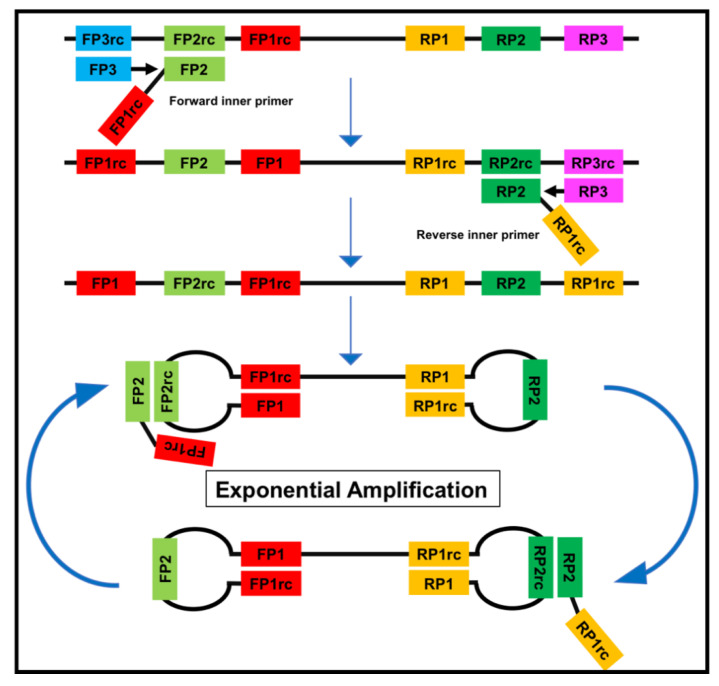
The loop-mediated isothermal amplification (LAMP) is based on a so called auto-cycling strand-displacement DNA synthesis using a strand-displacing DNA polymerase and two pairs of target-specific primers. The inner primers contain two sequences each corresponding to the sense and anti-sense (rc) strand of the target DNA, respectively, linked with a TTTT spacer. The inner primers initiate a complementary synthesis of the target DNA while the outer primers (FP1 and RP3) initiate a strand-displacement synthesis resulting in the release of single-stranded DNA linked by inner primers. The single-stranded DNA forms stem-loops by self-annealing of the corresponding sequences and acts as template for exponential amplification [23].

**Figure 3:**
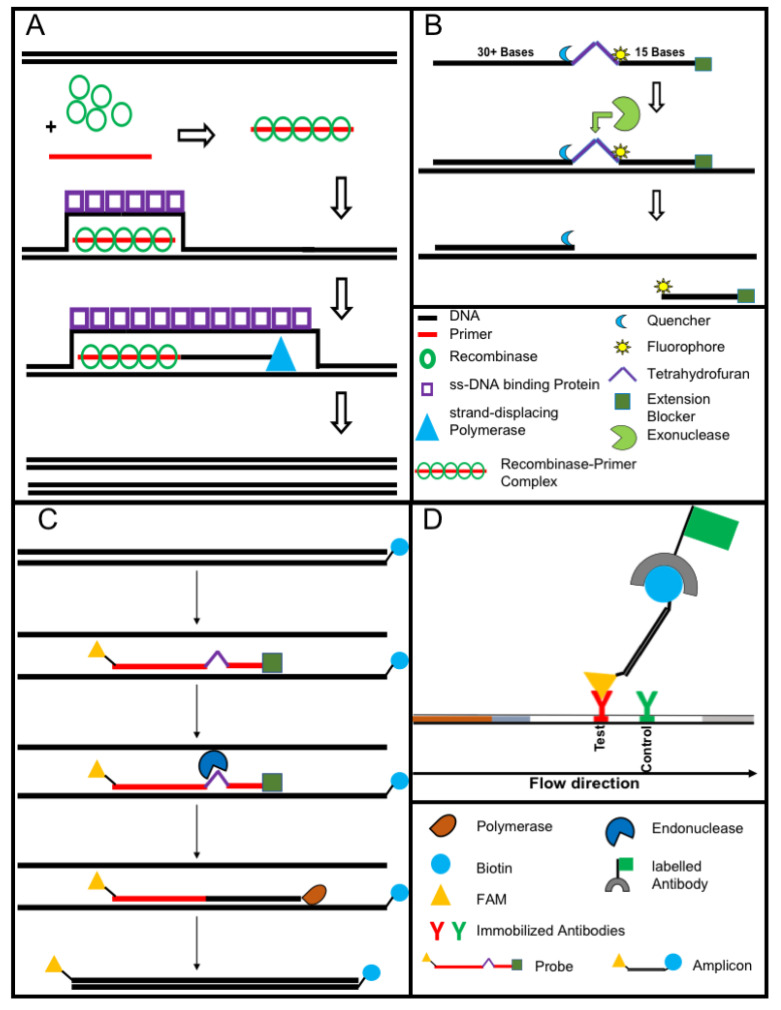
The recombinase polymerase amplification (**A**) employs a recombinase enzyme, a single-stranded binding protein, a strand-displacing (ss-DNA) polymerase, and a pair of target-specific primers. The primers form together with the recombinase enzyme the recombinase—primer complex which allows them to invade the double-stranded helix of the target DNA at the respective recognition site. The strand-displacing polymerase elongates the primers while the ss-DNA binding protein stabilizes the displaced strand in order to prevent self-annealing and ejection of the primers by branch migration. Real-time detection of amplification (**B**) is achieved by a sequence specific probe consisting out of a tetrahydrofuran abasic–site mimic (THF) flanked by fluorophore and quencher-labelled nucleotides as well as an extension blocker at the 3´ end. As the probe pairs with the complimentary sequence, a double-strand specific exonuclease slices the THF and the fluorophore is dissociated resulting in a signal [24]. Detection of RPA amplicon is in a lateral flow format (**C**,**D**). The use of a 5′end Biotin-labelled reverse primer in the RPA reaction leads to an antigenic-tagged reverse strand. When the 5-Carboxyfluorescein (FAM)-labelled probe binds to the complimentary strand a double-strand specific endonuclease slices the THF. The extension blocker is released and the remaining oligonucleotide can act as primer resulting in a FAM- and biotin-labelled amplicon (**C**), which can be detected in a lateral flow sandwich format (**D**, [25]).

Nucleic acid amplification methods have the advantage of being highly sensitive as opposed to immunological assays due to the amplification step. DNA is amplified using cycling methods such as polymerase chain reaction (PCR) or isothermal amplification. In contrast to the PCR, isothermal amplification assays have the advantage of employing a constant reaction temperature for the amplification. This offers more utility in the field due to the use of portable heat sources and shorter run time in comparison to the cycle driven PCR [21]. Moreover, most isothermal methods are known to be resistant to inhibitors existing in complex samples like blood [22]. Several different methods for isothermal amplification have been developed in recent years (Table 3). From these methods, the two most evolving techniques are the loop-mediated isothermal amplification (LAMP, Figure 2) and the recombinase polymerase amplification (RPA, Figure 3). All features of both methods are compared in Table 4.

### Equipment-Free Nucleic Acid Amplification

The implementation of equipment-free nucleic acid amplification at POC and PON is extremely challenging. Although there are equipment-free LAMP assays, they still need a heating source [26,27]. Nevertheless, to overcome the required power supply for the LAMP reaction, LaBarre et al. developed a portable heating cartridge based on exothermal-reaction and engineered phase-change material. Heat is generated by the reaction of calcium oxide and water. To keep the temperature in the optimal range, a fat-based compound with a specific heat capacity and melting point (65 °C) is applied. Beside LAMP, this cartridge is also suitable for other isothermal reaction like RPA or the nicking enzyme amplification reaction NEAR [28].

Nucleic acid lateral flow (immuno-) assays (NALFAs/NALFIAs) provided a simple and inexpensive read-out of the different amplification methods [29]. These assays are mainly based on the capture of tagged amplicons or detection of amplicons in a sandwich format [30]. The use of the lateral flow technique removes the need for sophisticated devices (e.g., a fluorescence reader). In some studies, isothermal amplifications were shown to operate on paper-based devices. While paper-based RCA [31] works on room temperature without equipment. RPA was also conducted equipment-free using the body heat for incubation combined with a lateral flow-based detection of the tagged amplicons. The principle of RPA lateral flow detection is shown in Figure 3C,D, [32].

The bottleneck of the molecular diagnostics is the nucleic acid extraction as high quality DNA is needed for both DNA amplification assays and sequencing. Moreover, the sensitivity of diagnostic method is questioned if the right extraction method is not implemented. Despite many breakthroughs and full automation of the DNA and RNA extraction, very few studies have tried to set up a field-applicable extraction procedure. The most common extraction procedure relies on the binding of the nucleic acid to a silica gel membrane after a lysis step using a cocktail of chaotropic salts and proteinases [33]. In such case, a high-speed centrifuge is necessary. Magnetic bead-based purification methods omit the need for a centrifugation step, but several pipetting steps are still essential and total hands-on time exceeds 60 min [34]. Recently, a simple one-step purification protocol was applied to the extraction nucleic acid of paratuberculosis from fecal samples [35], Leishmania from blood and skin [36,37], Ebola from saliva [38], and rabies from tissue samples [39]. This method combined detergents and heat for the lysis step and magnetic beads to capture the inhibitors. The total run time was 15 min and one pipetting was applied as washing was not required. A quick extraction method, which incorporates detergent, proteinases, and heat, was used to obtain the RNA from hepatitis C [40] and Ebola viruses [41]. Another approach based on two steps of alkaline lysis then neutralization was used for DNA collection from skin samples of Buruli ulcer patients [42]. Despite the availability of rapid extraction techniques, so far, they have not been widely used. Another important issue is the demand for non-invasive samples for diagnostics to avoid the hassle of the need for a highly-qualified person to collect complex samples like blood and cerebrospinal fluid at PON [43]. 

## 4. Metagenomic Diagnostics as a Tool for Outbreak Identification

Metagenomic diagnostics is the identification of pathogens after sequencing of genomic material collected from suspected cases [44]. This approach is based on the sequencing and identification of either all the nucleic acids present in the sample (shotgun metagenomics) or a particular group of previously generated amplicons (targeted-amplicon sequencing) [45]. 

The molecular detection through PCR or isothermal amplification of a pathogen requires a known target nucleic acid sequence. Thus, in an outbreak of unknown cause, selection of the right molecular assay upon the clinical signs is challenging and a novel pathogen or variants of known infectious agent may remain undetected [46]. Metagenomic diagnostics overcomes this problem by sequencing and identifying all of the nucleic acids within the sample and thereby preparing for simpler diagnostic testing. Also, information about epidemiology and transmission route can be achieved [47]. However, the performance of this method strongly relies on the used genome database and depth of sequence analysis as well as on the amount of generated reads [48]. The major drawbacks of metagenomic diagnostics are the high costs per sequencing sample and the relatively long turnover times [47].

**Figure 4:**
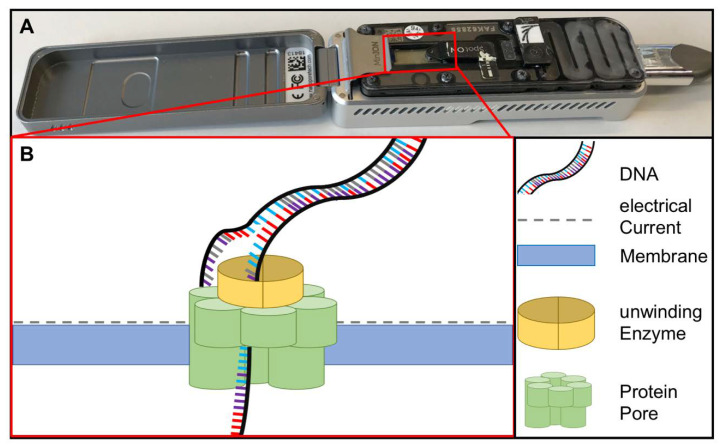
(**A**) MinION sequencing device (Oxford Nanopore technology, Cambridge, UK) and (**B**) the principle of nanopore sequencing. A protein pore is embedded in a membrane set under a certain electrical current. As the DNA molecule passes through the pore each nucleotide is recognized by the individual change caused in the voltage.

Sequencing methods require heavy and complex devices. However, the introduction of nanopore sequencing to the market by Nanopore Technologies allowed third-generation sequencing at PON due to small portable sequencing devices (Figure 4). The great utility of nanopore sequencing at PONf ne was proven by identification of pathogens direct from clinical samples [49] as well as during the surveillance for instance of Ebola and Zika outbreaks in West-Africa and South-America [50,51].

## 5. Why Is Every Method Important?

Immunoassays are an inexpensive, robust, and simple way to detect antibodies. Since different types of antibodies can be detectable after several days and up to several years in the blood [52], immunoassays represent an easy way for surveillance of previous and ongoing chronic infection. However, the identification of a disease based solely on antibody detection may lead to a false assumption about the diseased status of the individual. During persistent infections a detection of antigens and antibodies is normally possible over a long period of time; a latent infection (as a special case of persistent infection) is characterized by a very low antigen concentrations but high antibody level (for definitions see Table 5, [53]). In the latter case, nucleic acid amplification assays are needed as they can detect a low number of genomes with high specificity, which is especially helpful as for some chronic infections, the concentration of pathogens, antigens, and antibodies varies over the time [54]. Thus, the combination of immunological and genomic methods is necessary. On the other hand, as the immune response towards a disease starts a few days post infection and reaches the highest point around two weeks post infection [55], the detection of an acute infection is not possible solely based on antibodies. When the pathogen exists with a low concentration in the sample or is detectable for a short period of time, nucleic acid amplification assays are a good tool for diagnosis of such cases.

## 6. Solutions for Mobile Laboratories at Point-Of-Care and Point-Of-Need

The need for on-site diagnostic facilities was demonstrated during the West African Ebola outbreak as health systems of the affected countries were overloaded or not existing. Mobile laboratories were developed to enable diagnosis during outbreak situations. Their great advantage was their capacity to perform several diagnostic tests just as a central laboratory while being in the field at the site of an outbreak.

### 6.1. European Mobile Lab 

To overcome the problem of the long distances to the central diagnostic laboratories during the hemorrhagic fever outbreak in West-Africa, the European Mobile Lab Project established a moving laboratory unit in Nigeria, consisting of 27 boxes, each approximately 20 kg to 30 kg in weight. These boxes contained more than 400 equipment items needed to set up a fully-functional BSL3 or BSL4 diagnostic laboratory in a tent or in a local house. Minimum requirements were at least 28 square meters of space and a car for constant energy supply. European Mobile Labs enabled sample inactivation and preservation, molecular diagnostics using real-time PCR, antigen/antibody tests via ELISA and immunofluorescence assay as well as direct visualization of blood parasites using microscopy [56].

### 6.2. Mobile Suitcase Laboratory 

Originally developed for the field-applicable detection of avian influenza, the mobile suitcase laboratory was implemented in Guinea during the time of the West African Ebola outbreak. This PON diagnostics solution consists of two trolley cases, a solar panel, a power pack, and an optional glove box. One of the trolley cases was employed for nucleic acid extraction applying a reverse purification magnetic beads-based method. The second trolley case contained all instruments for the detection of the extracted nucleic acid via RPA or nanopore sequencing. The devices were placed on foam covered by a PVC top layer to be protected from shocks and to facilitate disinfection. The glove box can be used for sample disinfection and nucleic acid extraction in the case of BSL3 and BSL4 pathogens. All materials, kits, and reagents needed for nucleic acid extraction, isothermal amplification, and sequencing were stored in the suitcases. Due to the compact design, the mobile suitcase laboratory was easy to operate even in challenging surroundings [38,57].

### 6.3. Lab-In-Caravan

Another example for a mobile lab solution is the lab in a caravan. Based on the experiences made during the Ebola outbreak in West Africa in 2016, the Zika in Brazil Real Time Analysis (ZEBRA) project implemented two labs in caravan. These complete BSL 3 laboratories were set up in a trailer and used to collect and sequence samples of the Zika virus (ZIKV) using the nanopore sequencing technology along parts of the Brazilian east coast [58]. 

The Praesens Foundation developed a mobile laboratory implemented in a small truck [59]. The mobile laboratory contains a real-time thermocycler and a BSL3 glove box for sample preparation. The laboratory is air conditioned and can be hermetically sealed by the inside lower air pressure. For communication, Wi-Fi, mobile, and satellite connection are implemented. The lab has two batteries for power supply lasting more than 72 h. The truck has free bench spaces, which can be used for the operation of more diagnostic methods [60].

## 7. Lab-On-Chip Technology

The miniaturization and automatization of various laboratory workflows in one small apparatus were achieved through the lab-on-chip or microfluidics technologies. Bacterial concentration and recovery were conducted by combining ceramic membrane and tangential flow filtration [61]. In a size of 2 cm^2^, bacterial culture and quantification was accomplished by surface plasmon resonance and protein microarrays [62]. The advancement in the lab-on-chip allowed the integration of ELISA [63] and molecular assays (RPA [64] and LAMP [65]) in the form of a compact disc. Moreover, chips for improving the detection and accuracy of lateral-flow-based molecular assay were established based on oligonucleotide-linked gold nanoparticles [66]. The lab-on-chip facilitates controlled and fully automated diagnostic tests in a very short time. Nevertheless, the production cost is high, the design is a very complex procedure and not commercially available. 

## 8. Point-Of-Need Diagnostics in Epidemic Situations 

In the case of an emerging or new disease like coronavirus disease 2019 (COVID-19), the first step to identifying the causative agent is isolation and/or nucleic acid sequencing [67]. Upon identification of the severe acute respiratory syndrome coronavirus 2 (SARS-CoV-2), the most successful measurement was diagnosis and isolation of suspected cases since no vaccine or medicament were available. Molecular assays based on real-time RT-PCR is the main technology used worldwide to detect infected or asymptomatic cases [68]. Unfortunately, rapid antigen test performance was questionable since the viral load is very low in patient samples [69]. Many isothermal amplification technologies were developed to allow rapid case identification at local remote hospitals, in walk-through test centers and/or port-of-entry [23,70,71,72,73]. Regrettably, despite these methods having the same accuracy as PCR, the general acceptance of the method is very poor. Nevertheless, one of the big problems during epidemics is the rush in approving diagnostic tests and as a consequence, the assay efficiency is low [74,75,76]. There is a need for a unified assay validation regulation for emerging or novel pathogens. 

## 9. Conclusions

In the last three decades, more than 30 new infectious diseases have emerged, most of them zoonotic [77]. To prevent the pathogens from crossing species barriers or spreading globally, a reliable method for the identification of pathogens is needed urgently.

POCT and PONT are valuable tools for the diagnostics directly in the field and at the site of an outbreak. The big advantage of POC and PON diagnostics is the identification of pathogens directly at the site of the diseased person or animal with small turn-around times. So, the persons responsible for treatment can react more quickly according to the result. 

The use of POCT and PONT at airports, harbors, and quarantine stations can prevent the global spread of many infectious diseases due to international travel and trade. Furthermore, the sample volume needed for the tests is often smaller than in conventional laboratory tests. In addition, the use of POCT and PONT can lead to more adherence and optimization of the treatment regimen at the clinical site. Routine use of POCT and PONT can support the building of broad and comprehensive diagnostic infrastructure, especially in low resource settings, as the tests are mostly equipment-free and only small investments in local facilities are necessary (Table 6). 

The tests can be shipped easily to the location where they are needed and can overcome the pitfalls of low or demolished diagnostic infrastructures, especially in limited resource settings. These abilities make POCT and PONT an important instrument for the prevention of disease spread.

Unfortunately, current POCT and PONT are often designed for the detection of a single pathogen and do not have the diagnostic capabilities of laboratory tests. A platform suitable for the investigation of multiple pathogens is needed in order to identify the causative pathogen of an outbreak. Tests based on nucleic acid amplification can easily be adjusted to several pathogens. Once the outbreak causing pathogen is detected, a target-specific rapid test kit can be deployed to recognize the diseased individuals. 

Implemented in mobile laboratories POCT and PONT are a valuable tool not only for diagnostics in an outbreak situation but also for surveillance. This eases the implementation of functional diagnostics in a region with low infrastructure and helps to build up diagnostic capacity. 

## Figures and Tables

**Table 1:** Incubation Times of Selected Important Zoonotic and Animal Diseases.

Pathogen	Incubation Time (Days)
African swine fever virus	5–21
Suid herpesvirus 1 (Aujeszky’s disease)	2–10
Classical swine fever virus	2–14
Foot and mouth disease virus	2–14
Influenza viruses	1–4
Lumpy skin disease virus	4–28
Ebola virus	2–21
Marburg virus	2–21
Middle East respiratory syndrome virus	2–14
Rift valley fever virus	2–6
Severe acute respiratory syndrome virus	2–7
Hand, foot, and mouth disease viruses (Enterovirus)	3–6

**Table 2:** Important Pathogens Related With Epidemic Outbreaks After Natural Disasters as Defined by Brock et al. [9] in Comparison to Pathogens Listed by the WHO [10].

.	Brock et al.	World Health Organization
Bacteria	Methicillin-resistant *Staphylococcus aureus* *E.coli* Pseudomonas aeruginosa Methicillin-sensitive *Staphylococcus aureus* Enterobacter Klebsiella *Enterococcus faecalis* Coagulase-negative *Staphylococcus* *Streptococcus pyogenes* *Enterococcus faecium* *Serratia marcescens* *Streptococcus agalactiae* *Streptococcus viridans* *Acinetobacter baumanii* *Stenotrophomonas maltophilia*	*Vibrio cholerae* *E.coli* *Clostridium tetani*
Viruses	Human immunodeficiency virus Hepatitis B virus Hepatitis C virus West Nile virus Human T-lymphotropic virus Cytomegalovirus West-Nile virus Dengue fever virus Epstein-Bar virus Parvovirus B19 Chikungunya virus	Hepatitis A Hepatitis E Measles virus Dengue fever virus
		Other pathogens Plasmodia species Leptospira species Acute respiratory infections

**Table 3:** Different Isothermal Amplification Techniques.

Method	Reaction Temperature (°C)	Time to Result (min)	No. of Primers	Probe
Helicase-dependent amplification (HDA)	37	60	2	−
Rolling circle amplification (RCA)	37	90	1,2 or > 2	+/−
Recombinase polymerase Amplification (RPA)	39–42	3–10	2	+
Nucleic acid sequence-based amplification (NASBA)	41	90–120	2	+
Nicking enzyme amplification reaction (NEAR)	60	2–5	2	+/−
Loop-mediated isothermal amplification (LAMP)	60–65	60	6	+/−

+ use of probe for real-time detection possible; +/− use of probe possible, but usually not applied; − use of probe not possible.

**Table 4:** Features of Loop-Mediated Isothermal Amplification (LAMP) and Recombinase Polymerase Amplification (RPA).

Feature	LAMP	RPA
Isothermal	+	+
Visual read-out	+	
Portable heat source	+	+
Easy to implement in field applications	+	+
Fast result		+
Pair of primers		+
Simple assay design		+
Highly resistant to inhibitors		+
Long storage of reagents at room temperature		+

**Table 5:** Possibility of Antigen/Antibody Detection in Different Types of Infection [38].

Type of Infection	Detection of Antigens	Detection of Antibodies
acute	+	−
persistent	+	+
latent	−	+
chronic	+/−	+/−

+ detection possible, − detection not possible, +/− detection temporarily possible.

**Table 6:** Characteristics of Point-of-Need Diagnostic Test.

Feature	Condition
Portability	Easy to carry, transport, and use
Speed	Maximum 20–30 min
Equipment	No or one handheld device
Affordable price	1–5 USD
Accuracy	High: >90% sensitivity and specificity
Handling	Very simple or minimum manipulation
Storage and transport	Stable at room temperature
Production	Simple and fast manufacturing procedure and in bulk, preferred locally
